# OrganoidChip facilitates hydrogel-free immobilization for fast and blur-free imaging of organoids

**DOI:** 10.1038/s41598-023-38212-8

**Published:** 2023-07-12

**Authors:** Khashayar Moshksayan, Anirudha Harihara, Sudip Mondal, Evan Hegarty, Todd Atherly, Dipak K. Sahoo, Albert E. Jergens, Jonathan P. Mochel, Karin Allenspach, Janet Zoldan, Adela Ben-Yakar

**Affiliations:** 1 Department of Mechanical Engineering, University of Texas at Austin, Austin, TX USA; 2 Department of Biomedical Engineering, University of Texas at Austin, Austin, TX USA; 3 Department of Veterinary Clinical Sciences, Iowa State University, Ames, IA USA; 4 Department of Biomedical Sciences, Iowa State University, Ames, IA USA

**Keywords:** Biomedical engineering, Imaging, Lab-on-a-chip, Drug screening

## Abstract

Organoids are three-dimensional structures of self-assembled cell aggregates that mimic anatomical features of in vivo organs and can serve as in vitro miniaturized organ models for drug testing. The most efficient way of studying drug toxicity and efficacy requires high-resolution imaging of a large number of organoids acquired in the least amount of time. Currently missing are suitable platforms capable of fast-paced high-content imaging of organoids. To address this knowledge gap, we present the OrganoidChip, a microfluidic imaging platform that incorporates a unique design to immobilize organoids for endpoint, fast imaging. The chip contains six parallel trapping areas, each having a staging and immobilization chamber, that receives organoids transferred from their native culture plates and anchors them, respectively. We first demonstrate that the OrganoidChip can efficiently immobilize intestinal and cardiac organoids without compromising their viability and functionality. Next, we show the capability of our device in assessing the dose-dependent responses of organoids’ viability and spontaneous contraction properties to Doxorubicin treatment and obtaining results that are similar to off-chip experiments. Importantly, the chip enables organoid imaging at speeds that are an order of magnitude faster than conventional imaging platforms and prevents the acquisition of blurry images caused by organoid drifting, swimming, and fast stage movements. Taken together, the OrganoidChip is a promising microfluidic platform that can serve as a building block for a multiwell plate format that can provide high-throughput and high-resolution imaging of organoids in the future.

## Introduction

In recent years, organoid technology has emerged as an advanced in vitro model that can recapitulate the inherent characteristics of their corresponding organ tissue^1^. Interrogating various cell types and understanding the phenomena occurring within the organoid microenvironment requires high-content imaging (HCI). To achieve this goal, platforms are needed to enable high-resolution and high-throughput imaging of the samples in an automated, efficient, and fast manner. Flat-bottom devices that immobilize organoids in predetermined locations are essential for HCI.

Existing technologies only partially meet all HCI requirements. For example, multiwell plates are a conventional means for high-throughput culturing and screening of organoids (i.e., 96-, 384-, and 1536-well formats). These plates have wells with round U-, V-, or M-shaped bottoms (e.g., Elplasia® and Aggrewell™) that facilitate the formation of organoids (or spheroids) by self-assembly^2–4^. However, the deviation from flat-bottom in these wells introduces aberrations and does not allow high-resolution imaging of the organoids^5^. To combat this problem, users must transfer organoids to a multiwell plate with a flat-bottom glass surface for high-resolution and confocal imaging^2^. Even if the transfer of organoids is achieved successfully, the flat-bottom multiwell plates are susceptible to organoid drifting during fast stage movements or natural convection within the wells during imaging. These sample movements significantly hinder volumetric and high-resolution imaging of large organoids that require the capture of multiple field-of-views (FOVs) and z-stacks to image a whole organoid. Besides drifting, locating each organoid within an entire well is time-consuming and labour-intensive. This process requires changing between low and high magnification objectives and refocusing repeatedly to determine the organoid’s position. Another drawback of flat-bottom multiwell plates lies in the risk of some organoids being located at the edges of the wells, resulting in low-contrast and distorted images.

New microfluidic designs^6,7^, including the Pu.MA system^8^ and some other platforms, such as the Akura plates^5^ (InSphero AG), have overcome the aforementioned problems to some extent by placing organoids in smaller diameter wells (**~ **0.4–4 mm^4–8^). This feature keeps the organoids within a limited FOV and eliminates the need for locating them. Yet, such small wells allow only low-resolution imaging, as the organoids are not stationary within the FOV. Therefore, organoids can still move due to plate or fast stage movements, tilting-based perfusion within the microfluidic chip^6^, and any intrinsic sample movements as is the case in spontaneously contracting cardiac organoids.

The most common immobilization method involves embedding or forming organoids within hydrogels that enable their imaging, albeit at reduced resolutions^1,9^. Such hydrogel-based immobilization methods do not provide control over the location of the organoids within hydrogels. Most often, organoids are randomly distributed within the hydrogel at varying distances from the microscope objective and FOVs. This random distribution results in longer imaging times to locate the organoids within the hydrogel and requires long working distance objectives that commonly have limited imaging resolutions.

**Figure 1:**
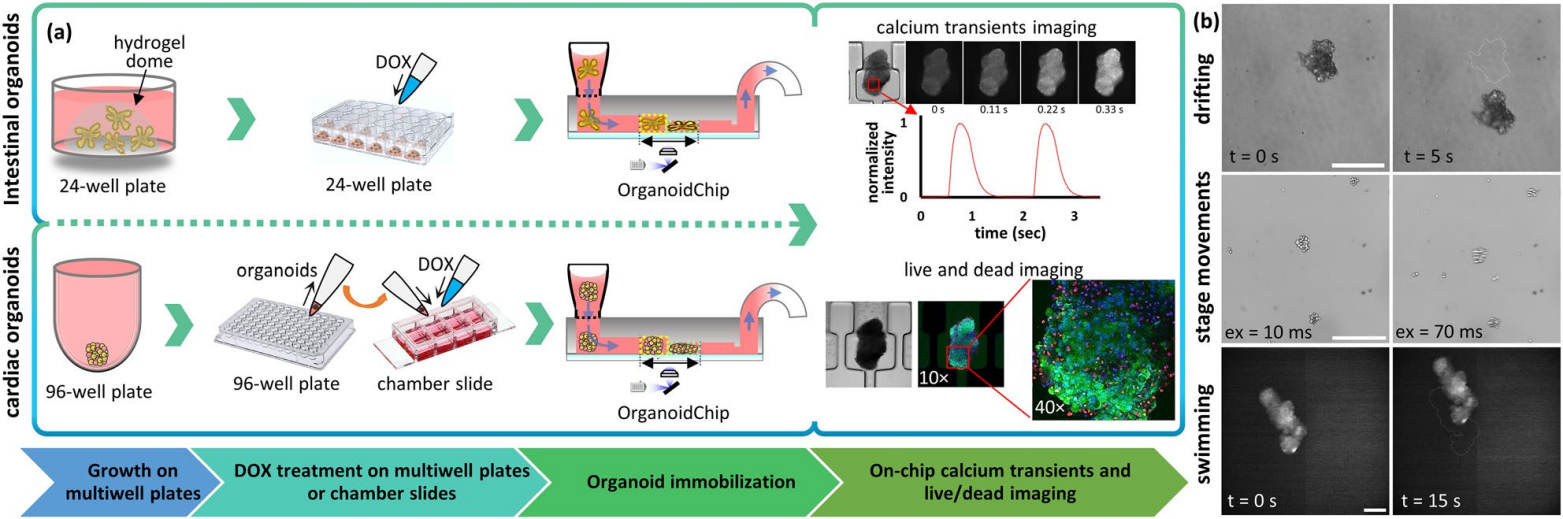
A flowchart describing the main steps of the OrganoidChip experiments (**a**), and various sources of image blurring and loss of data caused by lack of proper immobilization (**b**). (**a**) The organoids were cultured on multiwell plates and then exposed to DOX for 48 h. Subsequently, organoids were introduced to the OrganoidChip for immobilization followed by calcium transients (cardiac organoids) and live/dead imaging. (**b**) The figure has three rows, each one representing a mechanism that disrupts high-resolution imaging. The top row depicts drifting caused by natural convection within the well of a chamber slide over 5 s. The middle row illustrates how fast stage movements can cause blur when having short exposure times. Typically, larger momentums and shorter exposure times (shown as “ex” on the figure) result in greater blur. The bottom row shows how much a cardiac organoid can propel itself, or “swim”, in 15 s in a petri-dish during calcium transients imaging. Scale bars represent 200 µm.

To address these challenges, we present a new microfluidic imaging chip, called the OrganoidChip, with a novel design that does not require the use of any hydrogels for high-resolution imaging. The chip receives organoids from their conventional culture plates in which they were developed into organoids and treated with drugs, and holds them steadily within its immobilization chambers to facilitate end-point calcium transient and live/dead imaging (Fig. 1). We designed the chip with six trapping areas (TAs) in parallel, each consisting of two consecutive chambers: the staging chamber (SC) and the immobilization chamber (IC). The chambers were designed to slightly compress the organoids laterally and/or vertically to provide the necessary friction to fully immobilize organoids during imaging and to avoid blurring due to any organoid movements on the chip. The predetermined locations of the immobilization chambers accelerate the imaging process by eliminating the need for scanning across a large FOV to locate the organoids and changing between low and high magnification objectives during high-resolution imaging. As a proof-of-concept, we demonstrate the unique capabilities of the OrganoidChip by successfully immobilizing cardiac and intestinal organoids that were treated with a chemotherapeutic drug, Doxorubicin (DOX), at increasing concentrations. This platform enables image-based analysis of DOX treatment, demonstrating 50% inhibitory concentrations (IC_50_) values that are comparable to off-chip results.

## Methods and materials

### OrganoidChip fabrication

We fabricated the microfluidic chip by making a three-layered mold produced by photolithography of negative photoresists SU8-2050 and SU8-2100, followed by soft lithography of polydimethylsiloxane (PDMS). Three photoresist layers, having heights of 100, 190, and 260 µm, were stacked during fabrication, to provide channel heights of 100, 290, and 550 µm, respectively, in our device. Briefly, a 4-inch silicon wafer (STK9671-1, Nova Electronic Materials) was dehydrated on a hot plate at 120 °C for 30 min and installed on a spin coater. Depending on the desired layer thickness, we poured 4 ml of either SU8-2050 or SU8-2100 at the centre and spun. The wafer was carefully removed from the spin-coater and soft-baked. After cooling down to room temperature, the wafer was exposed to UV through the printed high-resolution mylar mask of the specific layer. The wafer photoresist was developed only after the first and third UV exposures (see Supplementary Table S1 for the quantities of time, temperature, etc., used in the fabrication). The mold was salinized using (TRIDECAFLUORO-1,1,2,2-TETRAHYDROOCTYL) TRICHLOROSIL-ANE (Gelest, Inc.) for 72 h to facilitate proper separation of the mold from the PDMS.

For the soft lithography, the base polymer and the curing agent were mixed at a 10:1 (w/w) ratio. The mixture was degassed in a vacuum chamber and was then slowly poured onto the mold, followed by oven-baking at 75 °C for 6 h. The PDMS was peeled off, punched for inlet and outlet tubings, and bonded to a #1.5 cover glass using oxygen plasma treatment to form the chip.

### Fluidic setup and its operation

**Figure 2:**
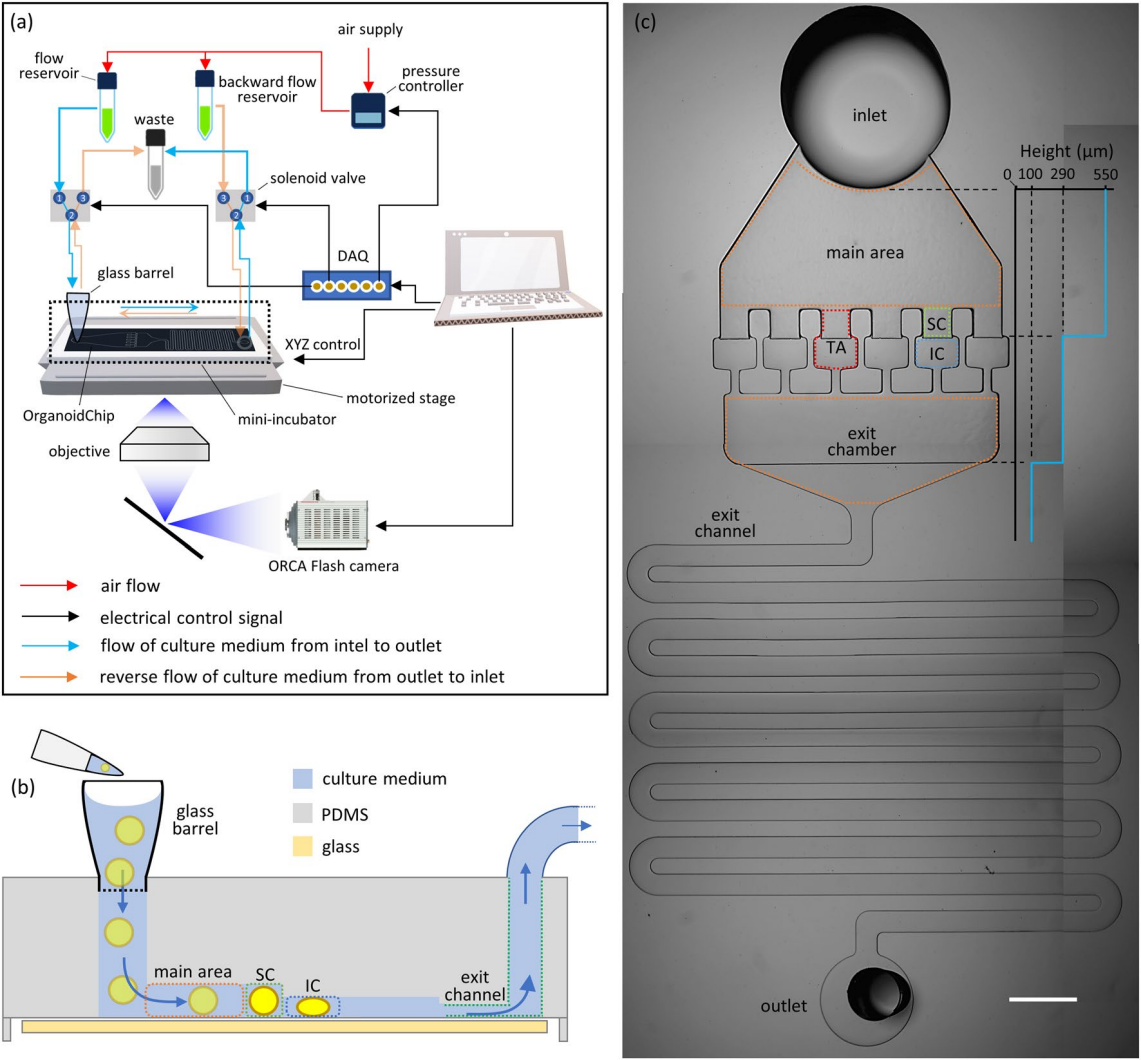
Microfluidic device design of OrganoidChip and its setup for immobilizing multiple organoids for blur-free imaging. (**a**) The microfluidic setup enabling forward and backward flow control (blue and orange, respectively) within the chip that was facilitated by creating a fluidic H-bridge using two solenoid valves. (**b**) A schematic of side view of the chip, demonstrating its operation. Organoids are pipetted into the glass barrel that is installed on top of the chip inlet, loaded into the main area, and immobilized in the trapping area (TA) within the staging chamber (SC) or immobilization chamber (IC) enabling blur free imaging. (**c**) A picture of the entire chip; its height changes and its various sections with dashed boxes of various colours, the main area (orange), SC (green), IC (blue), TA (red), exit chamber (orange), and the serpentine exit channel with a width of 315 µm, and a length of 71 mm. At the right side of the figure, a profile shows the height variations starting from the main area and extending into the exit channel. Multiple Images were stitched together to form the image of an entire chip. Scale bar represents 1 mm.

The Fig. 2a presents the microfluidic setup. The flow in the chip was regulated by an air pressure controller (ITV0010-3UBL, SMC) capable of delivering pressures between 0.01–1 bar. The regulator output was connected to two reservoirs filled with culture medium to establish a pressure-driven flow. The reservoirs and the chip were connected to two solenoid valves to create a fluidic H-bridge. With this design, we could reverse the flow direction within the chip at any time by controlling the solenoid valves using a DAQ card (NI USB-6009) and a LabVIEW program (version 13.0f2).

Prior to loading the organoids, the chip underwent several perfusion steps. The device was first perfused with DI water to eliminate air bubbles, followed by sterilization with ethyl-alcohol, and lastly with culture medium. A glass barrel (Precigenome LLC) was connected via Luer lock to the tubing that supplies culture medium, and was mounted on the chip inlet, serving as a nutrient reservoir. The glass barrel also served as an access point for loading the organoids and the fluorescent dyes onto the chip (Fig. 2a,b). To establish a hydrostatically driven flow within the OrganoidChip, which was needed when using an open-top glass barrel, we placed the waste reservoir 10 cm below the chip level. This height difference provided a flow rate of 16 µl/min in the chip. The hydrostatically driven flow was initiated by opening the solenoid valve that connected the chip outlet to the waste reservoir.

### Cardiac organoid culture and maintenance

CMV-GCaMP2 transfected human induced pluripotent stem cells (hiPSCs), a gift from Dr. Bruce Konklin, were used to generate cardiac organoids and visualize spontaneous intracellular calcium transients. hiPSCs were maintained in Complete Essential 8 Media (E8, StemCell Technologies) on vitronectin-coated culture dishes. Prior to cardiac differentiation, hiPSCs were seeded onto Matrigel-coated multiwell plates at a seeding density of 3.16 × 10^**5**^ cells/cm^**2**^. Upon reaching an 80% confluence, cardiac differentiation was initiated by WNT/ β-catenin pathway modulation^22^.

Spontaneously beating cardiomyocytes were harvested using Accutase (StemCell Technologies) 21 days after differentiation initiation. Cells were seeded at varying cell densities into ultra-low attachment round bottom 96-well plates (Nexcelom Biosciences). Next, seeded plates were centrifuged at 300 g for 5 min to facilitate cell aggregation. The organoids were cultured in suspension for 48 h in RPMI-1640 (Hyclone™) media supplemented with B-27 with insulin (Gibco™) and 10 µM ROCK inhibitor, to promote cell viability and organoid formation. Thereafter, media was replaced with media devoid of ROCK inhibitor and organoids were maintained in individual wells until they were transferred to chamber slides for DOX treatment followed by chip loading.

### Ethical animal use

The collection and analysis of intestinal biopsy samples from dogs were previously approved by the Iowa State University (ISU) Institutional Animal Care and Use Committee (IACUC-19–102; PI: Albert E. Jergens). All methods were performed in accordance with the relevant guidelines and regulations of IACUC as required by U.S. federal regulations (Fish, 2004). The study is reported in accordance with ARRIVE guidelines (https://arriveguidelines.org)^23^.

### Intestinal organoid culture and maintenance

We used canine intestinal crypts containing LGR5^+^ stem cells to facilitate the fast creation of mature intestinal organoids^9^. Canine intestinal crypts were obtained from endoscopic biopsies of healthy adult canines at Iowa State University (IACUC-22–050). Mature organoids containing villi, crypts, and lumen could be produced typically within 3 to 6 days after a passage^4,24^. At the time of seeding, canine intestinal crypts were suspended in Matrigel (Corning® Matrigel® GFR) and dispensed in 20 µl droplets onto a 24-well plate. Typically, every Matrigel pad contained 20 – 25 organoids. When organoids became larger than 400 µm, we employed a splitting ratio of 1:3. After a 10-min incubation at 37 °C and 5% CO_2_, 0.5 ml of the Complete Medium with Growth Factors (CMGF^+^) containing 10 µM ROCK inhibitor and 2.5 µM GSK3β inhibitor was added to each well and maintained for 48 h. Next, the medium was replaced with fresh CMGF^+^ without ROCK and GSK3β inhibitors and continuously refreshed every 48 h for 7 days. The organoids in the Matrigel pads were passaged and expanded on day 7.

To passage the organoids, they were first recovered from the Matrigel pads. Briefly, the Matrigel pads were dissociated by first removing the spent media and adding 0.5 ml of Complete Medium without Growth Factors (CMGF^−^) at 4 °C to each well to pipette the entire Matrigel pad until it was broken and separated from the well plate. Then, the suspension was centrifuged at 100 g for 5 min at 4 °C, and the supernatant was removed.

For passaging**,** 1 ml of TrypLE Express (Gibco, ThermoFisher Scientific) was added to the pellet and incubated at 37 °C and 5% CO_2_ for 10 min. Following centrifugation at 100 g for 5 min at 4 °C, the supernatant was removed and replaced with 5 ml of CMGF^**-**^ to stop the dissociation. An additional centrifugation step replaced the CMGF^−^ with the necessary amount of 4 °C Matrigel (depending on the number of cells). The suspension was dispensed onto a 24-well plate as 15 to 20 µl droplets. The plate was incubated at 37 °C and 5% CO_2_ for 10 min to allow Matrigel solidification; then, 500 µl CMGF^+^ was added to each well for further culture. The Matrigel pads were cleaned up 3 or 4 days after each passage to remove the dead and degenerative cellular debris, as well as very small organoids^9^. The clean-up process included all the steps described above for organoid recovery from Matrigel pads except for the TrypLE Express treatment.

Prior to DOX treatment, intestinal organoids were passaged 3 times and subsequently cultured for 7 days, with a clean-up performed on Day 5 to obtain the suitable organoid size and morphology. For chip loading, intestinal organoids were first recovered from the Matrigel pads, as described above. An additional step was required to fully dissociate any remaining Matrigel on the organoids. This step was essential to prevent any organoid agglomeration. Specifically, we added 1 ml of the Cell Recovery Solution (Corning Inc.) to the centrifuged pellet, pipetted a few times, and then incubated the recovered organoids at 4 °C for 30 min to completely dissociate any Matrigel that was still attached to the organoids. Following an additional centrifugation at 100 g for 5 min at 4 °C, the pellet with clean organoids was recovered in CMGF^**-**^ and transferred to a petri dish for loading into the chip.

### Doxorubicin treatment of organoids

We used 300 to 500 µm diameter organoids for treatment with DOX (0.1% (v/v) in DMSO; Supplementary Figure S1) for 48 h. Cardiac organoids were first transferred from round bottom 96-well plates to chamber slides (Ibidi Inc.) with a 170 µm thick glass coverslip to enable aberration-free imaging. Before DOX treatment, cardiac organoids were incubated overnight in 8-well chamber slides and imaged for brightfield and calcium transients. These measurements served as baseline for analysing the cardiotoxicity of DOX. Cardiac organoids were treated with 0, 0.1, 0.5, 1, 2, 5, and 10 µM DOX. Intestinal organoids were subjected to 0, 0.1, 0.3, 1, and 2 µM DOX within Matrigel pads on their native 24-well plate. The maximum concentration that the intestinal organoids were subjected to was slightly lower due to their higher sensitivity to DOX^25,26^.

### Cardiac organoids beating kinetics analysis

We performed 10-s-long, calcium time-lapse imaging of cardiac organoids pre- and 48 h post-DOX treatment to monitor the change in the beating kinetics of organoids in chamber slides (off-chip beating kinetics data). Then, the same organoids were transferred to the chip and allowed to habituate in the TAs for 100 min before on-chip imaging. During imaging sessions, cardiac organoids in the chamber slides and the chips were maintained at 37 °C in a humidified mini-incubator (TA-MI-20 × 46, Bioscience tools). Supplementary Figure S1 presents a detailed flowchart describing the entire DOX treatment and calcium imaging of the cardiac organoids on chamber slides and the OrganoidChip.

To analyse changes in cardiac organoids’ beating kinetics, regions within the organoid that demonstrated calcium transients were identified, and their average signals were monitored across time-lapse images. The beating waveforms, extracted from these regions, were used to estimate various beating kinetics parameters using an in-house MATLAB code. Specifically, we measured the beating rate (BR) and the ratio of the change in calcium fluorescence intensity to the baseline intensity (*ΔF/F*_*0*_). We also estimated the beating time percentage (*BT*_*75*_) and beating power (*FBT*_*75*_) defined by:

1 $$BT_{75} = \frac{{T_{75} }}{{T_{BR} }}$$

2 $$FBT_{75} = \frac{\Delta F}{{F_{0} }} \times \frac{{T_{75} }}{{T_{BR} }}$$

where ${T}_{BR}$ represents the average beating period of an organoid and ${T}_{75}$ represents the average duration of beating until the GCaMP fluorescence amplitude drops by 75% (Supplementary Figure S2). We then calculated the fractional changes ($\Delta \left(X\right)$) of various parameters for each individual organoid according to:

3 $$\Delta \left( X \right) = \frac{{X_{post\_tx} - X_{pre\_tx} }}{{X_{pre\_tx} }}$$

where ${X}_{pre\_tx}$ represents the pre-treatment and ${X}_{post\_tx}$ represents the post-treatment values of the specific parameter. Measuring pre- and post-treatment values required us to track individual organoids as they were transferred from the chamber slide to the chip by looking at their shape, area, and brightness.

### Live/dead staining, imaging, and analysis

We stained the trapped organoids on-chip using Calcein AM (Invitrogen™), Ethidium Homodimer-1 (EthD-1) (Invitrogen™, Waltham, MA), and Hoechst 33,342 (ThermoFisher Scientific Inc.) to label live cells, dead cells, and the cell nuclei, respectively. All dyes were administered at 2 µM, according to the manufacturers’ protocol, in culture media. The dyes were introduced into the chip through the glass barrel by replacing culture media with dye solutions and applying hydrostatically driven flow in an on–off cycle (1 min on and 10 min off) for a total of 50 min. We used a flow rate of 16 µl/min, which was sufficient to replace the entire chip volume of ~ 7.7 µl in less than 1 min. Prior to imaging, we perfused the chip with fresh culture medium for 2 min to remove any unbound dye and to minimize imaging background signal. We imaged the organoids using widefield and confocal fluorescence microscopies.

To control for the impact of the OrganoidChip, a subset of cardiac and intestinal organoids was stained and imaged off the chip (termed “off-chip”). Off-chip organoids were treated similarly with 2 µM dyes, followed by a 50-min incubation at 37 °C. The off-chip cardiac and intestinal organoids were imaged on 8-well flat bottom chamber slides and within the Matrigel pads in 24-well plates, respectively.

For viability analysis, we first created a mask, using the brightfield image, to define the boundaries of the organoids. We used the mask on the maximum intensity projection images to calculate the mean intensity of the live (*S*_*green*_) and the dead (*S*_*red*_) signals of the pixels within the mask and defined the viability ratio (VR) of each organoid according to:

4 $$VR = \frac{{S_{green} }}{{S_{green} + S_{red} }} .$$

For the DOX treatment experiments, we normalized the VR of each organoid by the average VR of the vehicle control organoids to obtain the viability percentage with respect to control.

### Image acquisition and processing

An ORCA-Flash4.0 V2 Digital CMOS camera C11440-22CU (Hamamatsu Photonics K.K.) was used along with an IX73 Inverted Microscope (Olympus) for widefield and fluorescence imaging. A PZU-2000 SERIES XYZ automated stage with an ASI MS-2000-WK multi-axis stage controller was used for automated imaging and positioning of the well plates and the chip during imaging on the inverted microscope. Time-lapse images were obtained at 100 fps to capture the calcium transients (10 × , 0.3NA objective, Olympus). The whole imaging setup was controlled using a homebuilt LabVIEW (NI, USA) program.

Confocal imaging of trapped organoids on-chip was performed (Leica TAS SP8) immediately after the live/dead imaging on the widefield microscope. The z-images of Calcein AM (excitation: 488 nm, bandpass filter: 493–547 nm), EthD-1 (excitation: 552 nm, bandpass filter: 557–784 nm), and Hoechst (excitation: 405 nm, bandpass filter: 410–483 nm) were captured with step sizes of 5 and 3 µm using the 10 × and 40 × objectives, respectively. All the image processing was performed with ImageJ (NIH).

### Statistical analysis

We used OriginPro (OriginLab Corporation, version 9.9.0.225) to perform statistical analyses, curve fitting for the dose–response assessments, and IC_50_ calculations. We calculated the *p* values for group comparisons using t-tests or *F* tests where *p* < 0.05 (*) and *p* < 0.005 (**) were considered significant, after verifying assumptions of normality and checking for the equality of variances. The sigmoidal Imax model and the regression parameters are reported in the Supplementary Eq. S1 and Table S2. The data points in the curves and tables are represented as mean ± standard error of the mean (SEM).

## Results

### Undesired organoid motion and image blurring in conventional culture formats

Conventional methods for imaging organoids prove challenging because of the random locations of organoids within the wells of a multiwell plate and their proclivity towards movement during imaging. First, natural convection or fast stage movements can cause bulk media flow, potentially resulting in an organoid drifting within or even out of the FOV. Organoid drifting can result in the capturing of blurred images, while additionally the organoid’s focal plane is constantly changing during its motion in the culture media (Fig. 1b, Supplementary Video S1). We demonstrated the blur caused by fast stage movements by first retrieving intestinal organoids from their native Matrigel, introducing them into a chamber slide, and capturing images with various exposure times (10 to 100 ms) immediately after each stage movement. The resulting images showed that larger organoids with higher momentums were subject to larger movements after the stage stopped and created non-negligible blurs in the images (Fig. 1b; for more details see Supplementary Figure S3). To eliminate this problem, one needs to lower the stage speed or add a waiting time for the organoids to settle before taking the next image which increases the total imaging time, especially for high-throughput assays.

On the other hand, organoids that spontaneously contract can move/swim within the FOV, resulting in blurred captured images during time-lapse imaging. This challenge is especially evident when imaging the calcium transients in our spontaneously contracting cardiac organoids, in which the fluorescing region of interest is subject to significant motion. Figure 1b and Supplementary Video S2 show a cardiac organoid that is propelling itself in culture media in a chamber slide, as evidenced by the bursts of motion it undergoes following its contractions. Monitoring the moving region of interest will require significant post-processing. More importantly, the lack of full immobilization may result in a sample drifting or propelling itself out of the FOV entirely, resulting in complete data loss. By contrast, we present Supplementary Videos S3 and S4 that show the brightfield and fluorescence videos, respectively, of a strongly contracting organoid that is remained immobilized in a trapping area (TA), demonstrating the OrganoidChip’s advantage over conventional imaging platforms.

### OrganoidChip design considerations

The OrganoidChip was designed to address the challenges associated with organoids movements through a unique geometry to facilitate immobilization of organoids in pre-determined locations. While immobilization of organoids prevents any movements, the known locations of immobilized organoids eliminate the laborious search for organoids during imaging.

The chip consists of four sections: (i) main area, (ii) TAs, (iii) exit chamber, and (iv) exit channel (Fig. 2c). The main area acts as a diffuser by expanding the flow from the chip inlet to the chambers, facilitating the distribution of organoids amongst the TAs as they enter the chip. Here, we demonstrated a chip design with 6 parallel TAs. This design ensures that each TA will trap only a single organoid. When one TA receives an organoid, its hydraulic resistance increases; therefore, the flow directs the other organoids toward the unoccupied TAs (movie S1). Each TA consists of a staging chamber (SC), followed by an immobilization chamber (IC) (Fig. 2c). The SCs have the same height as the main area to inflict minimal resistance during the initial organoid loading. Subsequently, the organoids move into ICs having reduced heights that allow a slight compression and flattening of the organoids. The IC width (650 µm) is larger than that of the SCs (430 µm) to account for any expansion that may arise from this vertical compression. The resulting static friction secures the organoids in place and renders them stationary during fast motorized imaging. The IC dimensions accommodate organoids across various sizes, provided that they can enter the IC. Organoids with diameters larger than the width of SCs commonly do not enter the ICs and are held in place by slight lateral compression of the SC side walls, still enabling immobilization for imaging.

ICs are connected to the exit chamber through a narrow channel that prevents organoids from escaping the ICs (Fig. 2c and Supplementary Figure S4). The exit chamber is connected to the exit through a 71-mm long serpentine channel with reduced height (100 µm). This design provides the necessary hydraulic resistance to control and maintain the low flow rates needed within the chip. We implemented the height reduction within the wide portion of the exit chamber to prevent small aggregates of cellular debris, which might escape the ICs, from clogging the exit channel (Fig. 2c and Supplementary Figure S4).

Multiple organoids housed within two adjacent TAs can fit within a single FOV of the 10 × objective (1.5 × 1.5 mm^2^), which facilitates the imaging of at least two organoids per FOV (with at least one organoid per TA). Because the TAs hold the organoids in place at known locations, the OrganoidChip eliminates the cumbersome task of repeated scanning across an entire well to locate the samples, proving beneficial for larger screens.

The OrganoidChip operates under pressure-driven flow, as opposed to using constant flow rates (e.g., a syringe pump), to ensure that the flow rate decreases when the hydraulic resistance in the chip rises (Fig. 2a). A pressure build-up can arise when all TAs are occupied by organoids, potentially exposing them to undesirably high shear stress in a flow rate-driven system. Therefore, we used pressure-driven flow that can adjust the flow rate within the chip as the TAs are loaded with organoids. Once all TAs are loaded with organoids, there will still be a small flow rate around the organoids to provide sufficient nutrient exchange.

### Organoid loading and chip trapping efficiency

**Figure 3:**
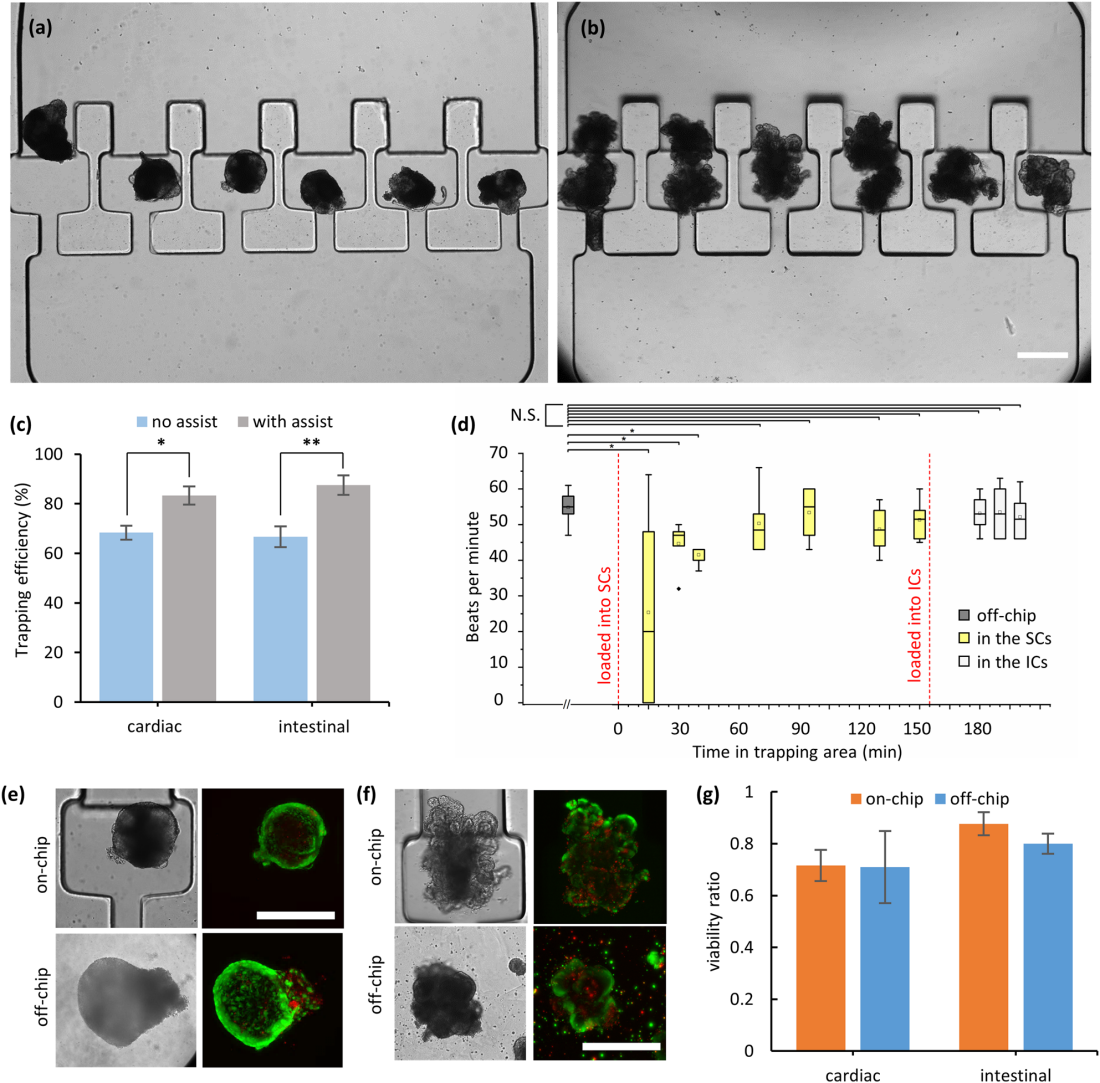
OrganoidChip performance characterization. (**a**, **b**) Representative images showing cardiac (**a**) and intestinal (**b**) organoids that were uniformly distributed between the TAs of the chip. (**c**) The trapping efficiency is reported as the number of filled TAs to the total number of TAs (i.e., 6). We performed 11 different sets of chip experiments for cardiac and 8 for intestinal organoids. Trapping efficiencies significantly increased when assisted loading procedures of flow reversal and chip titling were used (*n* = 6 TAs per chip). (**d**) Beating rate of the cardiac organoids as a function of time upon loading into the chip. The data shows no significant changes of the beating rates after 70 min (*n* = 6 organoids per condition). All comparisons made using a two-sample *t* test, accounting for equality of variances. (**e**, **f**) Calcein AM and EthD-1 staining of the off- and on-chip cardiac (**e**) and intestinal organoids (**f**) (20 × objective). (**g**) Viability ratios of the organoids obtained from 2 different sets of off- and on-chip experiments show no statistically significant differences (*p* = 0.40, non-chip = 18, and noff-chip = 15 for cardiac organoids; p = 0.36, non-chip = 8, and noff-chip = 9 for intestinal organoids). All scale bars represent 400 µm.

We developed a loading procedure that accommodates specific properties of each organoid type. For loading, the tube connected to the top of the glass barrel was removed and organoids were pipetted into the glass barrel. Depending on the size of available organoids, we loaded ~ 8 organoids on average. We transferred the cardiac organoids from the chamber slides to the chip by allowing them to settle to the base of the barrel without the presence of flow. Then, organoids were gently directed into the TAs using pressures up to 0.15 bar, corresponding to a maximum flow rate of ~ 13 µl/min per TA. Under these flow rates, we saw negligible or no cell shedding from the organoids, indicating that the imposed shear stress was not physically detrimental to the organoids. Figure 3a depicts an OrganoidChip with single cardiac organoid filling each of the six TAs.

Intestinal organoid loading proved more challenging as they were prone to adhering to one another during chip loading. Therefore, after Matrigel digestion in their native 24-well plate, intestinal organoids were transferred into a petri-dish and then pipetted one by one into the glass barrel while applying a hydrostatically driven flow. When an organoid gravitated to the bottom of the barrel and entered the chip, the flow directed the organoid to an empty TA. The organoids were constantly loaded one after another until all the TAs were filled with at least one organoid (Fig. 3b).

Figure 3c shows the trapping efficiency of organoids to be 68% on average during the initial loading in the device. We tested another chip with a larger number of TAs and found that the organoids did not distribute uniformly among the TAs due to the chip’s large width, resulting in low trapping efficiency^27^. Therefore, we decreased the overall width and the tapering angle of the main area by reducing the number of TAs to six, that increased the overall trapping efficiency. Occasionally, two or more organoids entered a single TA, leaving some other TAs empty. We implemented an assisted loading strategy to improve the trapping efficiency and reduce the risk of multiple organoids being trapped in a single chamber. After the initial loading, we reversed the flow to redirect the organoids into the main area, laterally tilted the chip to uniformly redistribute them amongst the TAs and resumed the forward flow to guide organoids into the empty TAs. Using this assistive loading procedure, the average trapping efficiency for both intestinal and cardiac organoids significantly increased to 85% (Fig. 3c).

### OrganoidChip does not affect the beating rate of cardiac organoids

To study the effect of the chip flow dynamics and the loading process on the functionality of cardiac organoids, we measured the changes in organoid beating rates using time-lapse imaging. Their average native off-chip beating rates were 55 beats per minute, as measured in 96-well plates using the BioTek Cytation 3. When the organoids were subsequently transferred into the OrganoidChip and distributed amongst the SCs, their beating rates initially decreased because of pipetting, temporary changes in temperature, and exposure to flow during loading. Yet, within 70 min of habituation inside the mini-incubator, with no flow in the chip, the organoids’ beating rates showed no statistically significant differences from their native values (Fig. 3d). During the following 80 min in the SCs, their beating rates remained unchanged.

We then moved the organoids into the ICs. Even after 30 min of compression in the ICs, the organoids’ beating rates were similar to those measured off the chip. To further investigate the isolated effect of compression on cardiac organoids, we performed a separate experiment on a petri-dish. Specifically, we compressed the organoids to a height of 230 µm using a cover glass and monitored their beating rates over time (Supplementary Figure S5). The results showed that the beating rates of compressed organoids approached pre-compression levels in less than 20 min, consistent with our on-chip observations.

### OrganoidChip does not affect the viability of organoids

Next, we tested if loading and flowing organoids into the chip affected their viability. We stained and imaged organoids both off and on the chip. The organoids imaged off-chip served as the controls.

The Fig. 3e,f show representative images of live and dead stained healthy organoids. The viability ratios, calculated by applying Eq. 4 on live/dead images, show no statistically significant differences between the off- and on-chip organoids (Fig. 3g). These results indicate that the chip loading and immobilization processes have no notable adverse effects on organoid viability. Additionally, these results highlight that our device enables staining in addition to imaging of organoids. Despite the additional processes that the intestinal organoids were subjected to, such as the pipetting and resuspension processes required for dissolving the Matrigel for on-chip imaging, their viability ratios are not significantly different than those imaged off-chip. These results further confirm that the additional steps required for chip-loading are also not detrimental to the cells.

### Dose–response to Doxorubicin treatment using live/dead assay

**Figure 4:**
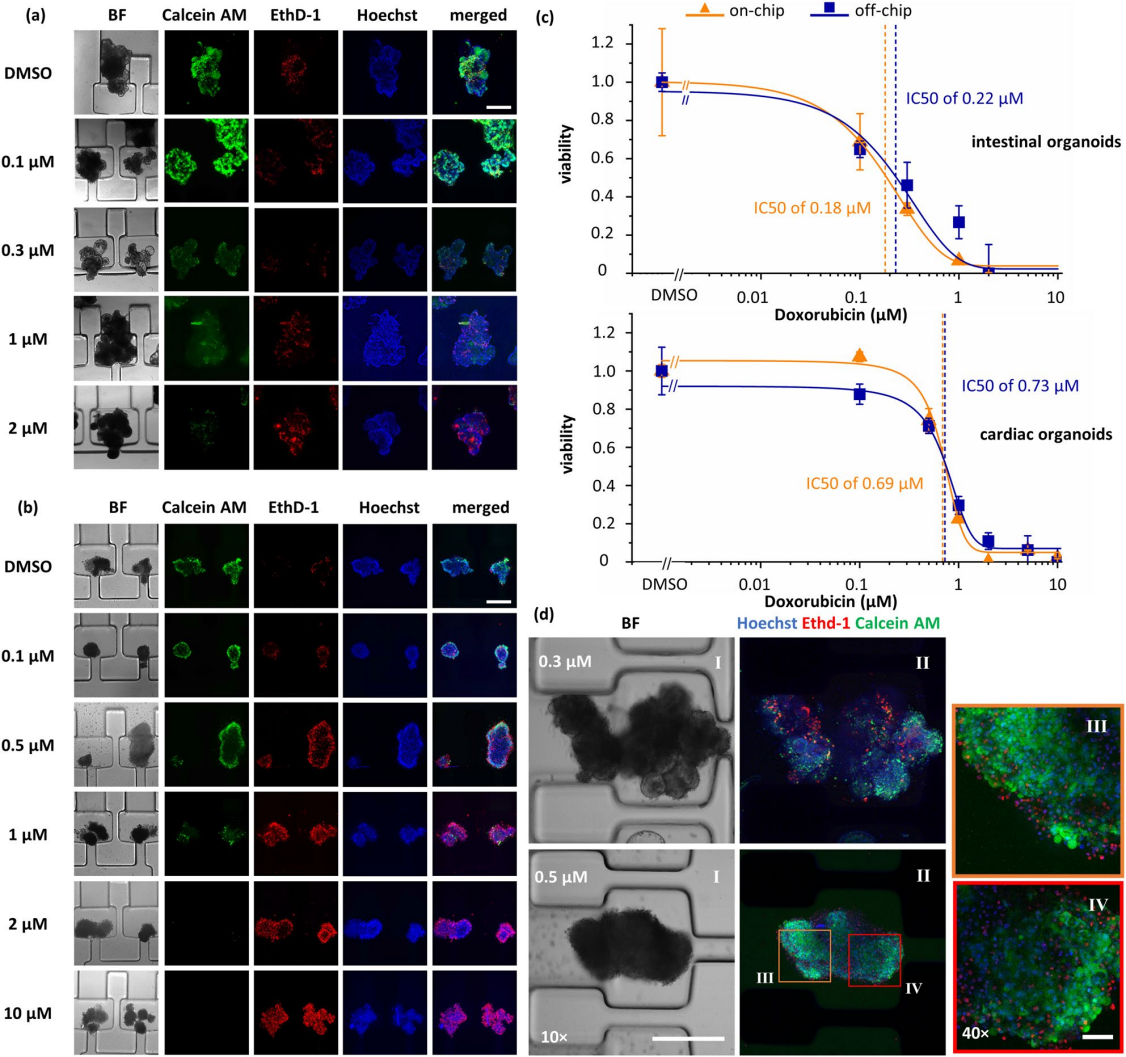
Dose-dependent viability analysis of the intestinal and cardiac organoids after 48 h of DOX treatment. Example images of the stained intestinal (**a**) and cardiac organoids (**b**) in the OrganoidChip. The intestinal organoids treated with 0.3 and 2 µM concentrations were imaged in the chips with shorter connecting channel design. (**c**) The viability curves for the off- and on-chip live (Calcein AM) and dead (EthD-1) staining results. The IC50 for the off- and on-chip conditions are 0.22 and 0.18 µM for the intestinal and 0.73 and 0.69 µM for the cardiac organoids, respectively. The data points are represented with an S-curve fit (n ≥ 5 organoids per condition); (**d**) brightfield (I) and maximum intensity projection (II) of the confocal z-images of organoids treated with 0.3 µM (intestinal organoid) and 0.5 µM (cardiac organoid) concentrations of DOX stained with Hoechst (blue), EthD-1 (Red), and Calcein AM (Green) (10 × objective); The orange (III) and red (IV) regions of the cardiac organoid were re-imaged using a 40 × objective and their maximum intensity projections are shown in the sub-images III and IV. Scale bars for 10 × images (in figures a, b, and d) and 40 × images (in figure d) are 400 µm and 100 µm, respectively.

We evaluated the performance of the OrganoidChip in assessing the dose-dependent toxicity response of organoids using live and dead imaging. Figures 4a,b present representative brightfield and live/dead fluorescence images of organoids treated with DOX at varying concentrations. Most organoids were successfully immobilized in the ICs. Since intestinal organoids were more prone to agglomeration, regardless of DOX concentration, occasionally two or more of them adhered to each other and remained as large clumps in the SCs (0.1 and 1.0 µM treatment cases shown in Fig. 4a). At high DOX concentrations, cardiac organoids were significantly smaller in size (< 350 µm) and appeared to adhere to each other, as can be seen in the 10 µM case of Fig. 4b. We speculate that dead cells shedding off the organoids surface (observed during off-chip DOX exposure) contributed to size reduction while changes in the surface properties and presence of free DNA from the dissociating cells caused organoid agglomeration. Having multiple organoids in the TAs (in ICs or SCs) did not adversely affect the chip functionality. Organoids were successfully imaged while immobilized in either chamber of the TAs.

The live/dead dose–response curves were obtained by fitting a sigmoidal Imax model (S-curve) to viability data points (Fig. 4c). Overall, the shape of the fitted S-curve was steeper for cardiac organoids vs. intestinal organoids. The viability starts dropping in the 10 s of nM range for the intestinal organoids while the drop begins in the 100 s of nM for cardiac organoids, implying that intestinal organoids are more sensitive to DOX treatment. For example, cardiac organoids treated with 0.1 µM DOX experience marginal effects on cell viability and these organoids exhibiting nearly the same viability as the DMSO-treated condition. On the other hand, treating intestinal organoids with the same DOX concentration reduces their viability to ~ 65%. At higher DOX concentration treatments, such as 2 µM, the viability of both types of organoids significantly drops to below 10%.

Based on these curves, we determined that the off- and on-chip DOX IC_50_ values are 0.22 and 0.18 µM for the intestinal organoids, and 0.73 and 0.69 µM for cardiac organoids, respectively (Supplementary Table S3). These results further indicate that intestinal organoids are more sensitive to DOX treatment than cardiac organoids, showing toxicity at lower concentrations. The fitted dose–response curves for the off- and on-chip tests are not statistically different (Supplementary Table S4), indicating that the loading, immobilization, and staining processes on the OrganoidChip do not alter the viability results for the DOX-treated organoids.

In the literature, there is a very limited number of studies presenting viability-based IC_50_ values for DOX-treated intestinal organoids using human-based cells and none for canine-based organoids One of human-based studies analysed images (4 × objective) of cells stained with Phalloidin-FITC and Hoechst to find cellular viability^28^. We estimated their IC_50_ values to be lower than 1 µM, comparable to our results. Viability analysis of DOX-treated cardiac organoids has been reported using different methods such as the absorbance-based assays^29^, live/dead fluorescence staining^3^, flow cytometry of cells dissociated from organoids^30^, and beating kinetics parameters (i.e. beating rate, area change, etc.)^13,31^. However, many of these studies did not test over enough DOX concentrations to generate exposure–response curves and, ultimately, could not estimate any IC_50_ values. Among those studies that reported IC_50_ values, they relied on beating parameters. For example, Richards et al. reported an IC_50_ value for cardiac organoids exposed to DOX for 48 h as measured from the fractional area change of the organoids during beating. They found an IC_50_ of 0.41 µM, comparable to our findings.

To show the capability of the OrganoidChip in enabling higher-resolution imaging, we used confocal microscopy for several organoids immobilized on the chip. Representative images show improved optical segmentation and the ability to resolve single cells within an organoid (Fig. 4d). The co-localized EthD-1- and Hoechst-stained nuclei are resolvable and can potentially be used to increase the accuracy of viability measurements. Future implementation of 3D-segmentation using AI-assisted algorithms in the analysis pipeline can provide more accurate estimations of cellular viability in larger screens.

### Calcium transience imaging revealed dose-dependent changes in beating kinetics parameters of cardiac organoids

Next, we measured the effect of DOX treatment on the beating kinetics of cardiac organoids. To do this, we relied on calcium fluorescence imaging, as it has been shown to be a good approximation of the cardiomyocytes’ action potentials^32^. Calcium imaging proved beneficial for beating and contraction parameters since smaller beating portions cannot necessarily be detected from brightfield images, particularly when organoids have been compromised as a result of drug treatment.

When assessing drug effects, we observed some degree of variability in the spontaneous contractile behaviour and beating kinetics between cardiac organoids. Such variability often skews any averaged parameter value across organoids and does not reflect the effect of the treatment conditions on organoid health. To address this challenge, we tracked each individual organoid’s beating off- and on-chip. The drug-induced functionality results are therefore reported as averages of fractional changes of each individual organoid’s beating kinetics parameters, measured at 48 h post-treatment, on both the chamber slide and on the chip, relative to its pre-treatment value (Eq. 3).

**Figure 5:**
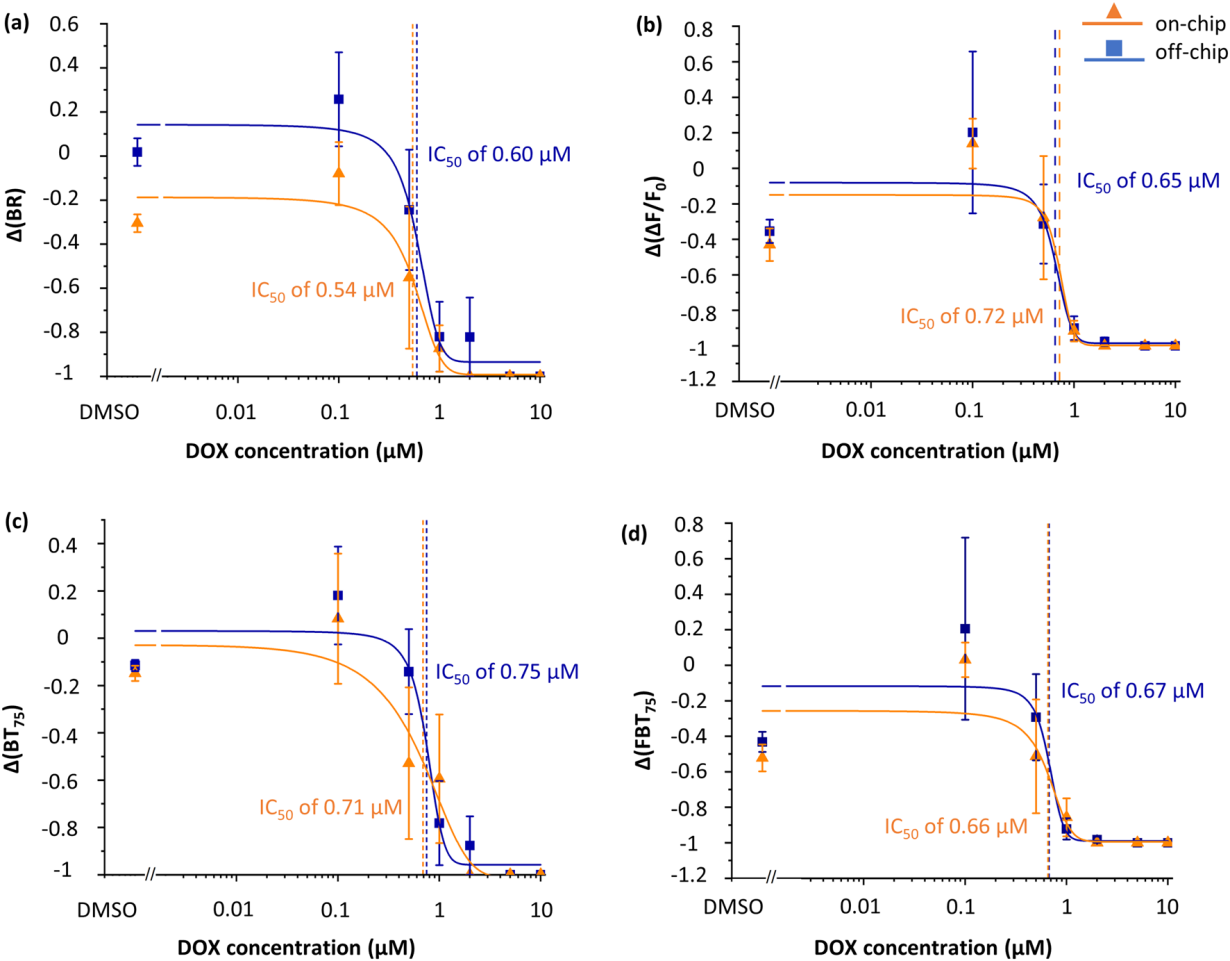
Dose–response S-curves of various beating kinetic parameters of cardiac organoids based on their fractional changes (i.e., Δ(X)). We present the curves of the fractional changes for the off- and on-chip beating rates (BR) (**a**), *ΔF/F*_*0*_ (**b**), the beating time percentage (*BT*_*75*_) (**c**), and the beating power (*FBT*_*75*_) (**d**). We fit an S-curve to data and obtained IC_50_ for the off- and on-chip conditions as summarized on the plots. Off- and on-chip curves as well as curves of different parameters compared well with each other with no statistically significant differences (*F* test). *n* ≥ 5 organoids per condition.

In assessing cardiotoxicity, we studied the well-reported beating kinetics parameters, *BR* and *ΔF/F*_*0*_. Additionally, we propose two new parameters: the beating time percentage (*BT*_*75*_) and the beating power (*FBT*_*75*_) to evaluate less-understood aspects of organoid beating kinetics (Eqs. 1 and 2). By fitting S-curves to the dose-dependent responses of organoids, we estimated IC_50_ levels of off- and on-chip conditions for each of these parameters (Fig. 5).

Interestingly, all IC_50_ levels estimated from beating kinetics parameters measured on-chip are nearly identical to their off-chip counterparts and to those reported in the literature (Fig. 5 and Supplementary Table S3)^31^. Additionally, the off- and on-chip S-curve fits are not statistically different (*F* test, Supplementary Table S4). Taken together, these results indicate that toxicity levels obtained from on-chip beating analyses are similar to off-chip measurements, further demonstrating that the OrganoidChip does not introduce bias when assessing organoid viability or functionality. Furthermore, the estimated dose–response curves across all the beating kinetics parameters are similar to each other (F-test, Supplementary Table S5). Moreover, their resulting IC_50_ values are within the bounds of the standard error of the IC_50_ values obtained from the viability analysis (Supplementary Table S3). These results indicate that calcium imaging can be used interchangeably with fluorescence live/dead imaging to obtain IC_50_ values.

### OrganoidChip facilitates fast imaging

We developed a LabVIEW program to control the camera and our widefield microscope stage for fast and automated imaging and stage movements. The program moves the stage across three FOVs on the chip, each containing two TAs (corresponding to two organoids), to image the six TAs with our high-speed camera. According to Table 1, we achieved imaging times of 10.4 and 70.0 s for on- vs. off-chip calcium transients imaging and 5.5 and 89.5 s for on- vs. off-chip live and dead imaging of two cardiac organoids. Eliminating the required times for finding an organoid in a well, changing to high-NA objective, and locating the correct ROIs and focal planes for the on-chip imaging, significantly reduced the imaging time (Table 1). The measurements show that the OrganoidChip is a promising tool for fast imaging to accelerate drug toxicity assessments if designed in a higher throughput manner, as discussed later in this manuscript.

**Table 1:**
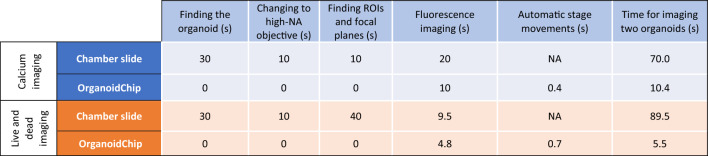
Time required for each step performed on a chamber slide and on the OrganoidChip for two cardiac organoids. We present the time for imaging two organoids because two TAs could be imaged within a single FOV on the chip using a 10 × objective (*n* = 3). The first 3 columns represent time consumed in locating the organoids. For the times required for introducing and removing the dyes, setting camera parameters, and bright-field imaging, refer to the Supplementary Table S6.

## Discussion

Devices that can provide robust organoid immobilization for high-resolution imaging that can be scaled up to a high-throughput configuration are lacking. High-throughput and high-resolution imaging requires organoids to be fully immobilized, which can be achieved by embedding organoids within hydrogels. However, using hydrogels has limitations such as random distances of organoids from the objective. To overcome this challenge, Cambra et al. developed a three-layered hydrogel sandwich for intestinal organoid culturing^10^. Using standard 12-well plates, organoids were grown in a narrow range of distances from the plate bottom. Others devised an immobilization method for brain organoids by embedding them in a hydrogel and restricting their heights between a cover glass and either a porous membrane^11^ or a 3D-printed plate insert^12^. These methods eliminate organoid drifting, and thus allow high-resolution and time-lapse imaging over multiple days. However, they also require cumbersome device fabrication and organoid loading and, most significantly, do not solve the issues related to the distribution of organoids in random FOVs, which may not be conducive to high-throughput formats.

Alternatively, organoids can be cultured on top of micropatterned PEG and Matrigel layers to produce immobilized organoid-like structures^13^. Although this method renders the organoids in a fixed FOV and is scalable to high-throughput culture, it only generates partial three-dimensional organoids, which are limited in their capability of emulating organ structure and function^14^.

The incorporation of organoids with microfluidic technologies has created useful platforms that can implement standard drug treatments^15^ with sophisticated assays^16,17^ to facilitate sample monitoring and end-point analysis through imaging^7,18^. Though current microfluidic devices can probe many intricate aspects of organoids, many are not scalable to high-throughputs due to cumbersome processes such as sealing or assembling the chip after the introduction of the organoids^11,19,20^, and having design features that are difficult to fabricate or are not conducive to cost-effective fabrication methods (e.g., injection molding). Some state-of-the-art microfluidic devices have managed to facilitate the immobilization of organoids using hydrogels^17,20,21^. Despite the outstanding capabilities of these devices, finding the desired FOV and focal plane, in most of them, remains a challenge for high-resolution imaging. Aeby et al. addressed the random dispersion of organoids by utilizing a hydrogel hanging droplet culture within a microfluidic device that could be perfused for over nine days^21^. Although the device performed outstanding assays using colon and liver spheroids, these solid hydrogels may not be a suitable bed for studying certain types of organoids, such as spontaneously beating cardiac organoids. More importantly, the addition and removal of the fluorescent dyes from hydrogels solely rely on dye diffusion, which can be a relatively slow process regulated by hydrogel porosity and crosslinking density.

Even when loaded with organoids, the OrganoidChip is devoid of any hydrogel materials, avoiding any previously discussed complications associated with hydrogel-assisted methods that lead to slower and smaller-scale tests. To further enhance the imaging speed and implement high-throughput imaging, our chip can serve as a building block to design a 96-well plate, in which each well corresponds to an OrganoidChip, similar to the high-throughput vivoChip platform that we previously developed^33^. We speculate that the time saved by using such multiwell chip in an automated fashion will facilitate large-scale screening that can be performed in a short amount of time. Our estimations indicate that the sum of imaging times for calcium transients and live and dead imaging of 96 × 6 organoids are 2 h versus 25 h for the hypothesized 96-well OrganoidChip plate versus six conventional flat-bottom 96-well plates with one organoid per well, respectively (see Supplementary Table S6). An order of magnitude reduction in time was made possible by imaging two organoids at a time on the chip and removing the time associated with finding organoids within a well or chamber slide, introducing and removing dyes, and redetermining the organoids’ position after dye incubation. This saved time renders high-throughput screening feasible for time-sensitive tests such as clinical applications involving personalized medicine, demonstrating our platform as a crucial and enabling technology for the organoid community and beyond.

Finally, we emphasize that imaging calcium transients of spontaneously contracting cardiac organoids proves challenging as the fluorescing region of interest is subject to significant motion when imaging off-chip (Supplementary Video S2). Lots of post-processing are necessary for monitoring the same region of interest using cross-correlation metrics of the organoid’s position if the organoid is still within the FOV. If the organoid propels itself outside of the FOV, the calcium transient data is commonly lost in conventional imaging methods. As we foresee the OrganoidChip as a building block for a higher-throughput device, minimizing post-processing steps to account for organoid movements during timelapse imaging will prove to save time and processing power.

## Conclusions and future directions

We developed a microfluidic device, the OrganoidChip, that enables immobilization and staining of organoids all in one device for fast and blur-free imaging that minimizes sample loss during assay administration. This device overcomes the limitations of existing HCI platforms for organoid imaging and is compatible with both high-resolution widefield and confocal microscopies. We demonstrate these unique capabilities of the OrganoidChip by performing HCI on DOX-treated intestinal and cardiac organoids for dose-dependent toxicity assessment.

The unique design of the OrganoidChip allows immobilization of organoids to prevent undesired movement during fast motorized imaging, thus eliminating the risk of an organoid drifting out of focus and FOV. Pre-determined locations of the immobilization chambers eliminate the cumbersome and time-consuming task of searching across an entire well or hydrogel pad to locate organoids. Our chip is also advantageous for volumetric imaging of organoids. For larger organoids, the limited height of the TAs allows for imaging using a fewer number of z-slices. Specifically, the crypts and villi structures inherent to intestinal organoids require many optical sections to completely image the organoid morphology (Supplementary Figure S6). Taking fewer images reduces the required time and minimizes data storage space.

We successfully loaded, immobilized, stained, and imaged organoids with the OrganoidChip and showed that these processes do not affect the viability or functionality of the organoids. Specifically, we studied cardiac and intestinal organoids that were exposed to DOX, a standard chemotherapeutic drug, for 48 h in a dose-dependent manner. The effects of DOX toxicity on the viability of both organoid types and functionality of cardiac organoids were found to be nearly identical between the off- and on-chip tests. The estimated IC_50_ values were similar to published data from the literature, especially those performed using image-based analysis^4,28^. These studies have used low-resolution microscopy (i.e., 4 × objective) to reduce image blurring and eliminate time-consuming scans for locating individual organoids within a well for viability assessment. Higher resolution imaging (i.e., ≥ 20 × confocal imaging) might potentially provide more accurate and sensitive detection of drug-induced cytotoxic effects which can be implemented using the OrganoidChip.

Due to the organoids’ tendency to agglomerate and the stochasticity of organoid distribution among TAs, the loading process needs manual tilting and flow reversal to achieve high trapping efficiency. Although such manual interventions are feasible for small-scale studies, they are not practical for large-scale studies using a multiwell chip. This challenge can be addressed by tilting the chip steadily on an automated rocker during the loading process to promote a better organoid distribution among the TAs. Otherwise, we can also design the chip with a single TA per well, in a 384-well plate format, to eliminate organoid agglomeration since there will be only one organoid per chip and, thus, the trapping process will be deterministic.

The dimensions of our device are designed to be compatible with cost-effective fabrication methods, such as hot embossing and injection molding. These features can be replicated into a hot embossing mold by means of elastomeric transfer from the SU-8/Si wafer. The minimum feature size of the microchannels is on the order of ~ 0.1 mm to ensure that the chip’s features are compatible with CNC micro-milling, making it an ideal candidate for scaling up production via injection molding, in the future.

Future versions of OrganoidChip may incorporate wells for culturing organoids, specifically cardiac organoids, within the imaging device. The automatic introduction of organoids into the trapping areas will significantly reduce the challenges associated with pipetting during the organoid transfer process^33^. Cultured organoids can also be optically cleared, fixed, immunostained, imaged on chip, and retrieved from the chip for further analysis.

By and large, our data indicate that the OrganoidChip eliminates many of the current technologies’ shortcomings for imaging organoids. Furthermore, the recent FDA modernization Act 2.0, approving the use of organoid data for novel drug IND submission instead of in vivo animal testing, highlights the potential of the OrganoidChip in the preclinical evaluation of therapeutic drug candidates.

## Supplementary Information

Supplementary Information 1.

Supplementary Video 1.

Supplementary Video 2.

Supplementary Video 3.

Supplementary Video 4.

## Data Availability

The datasets generated and analysed during the current study are available from the corresponding author on reasonable request.
