# Supplementary material for: OrganoidChip facilitates hydrogel-free immobilization for fast and blur-free imaging of organoids
Source: Sci Rep. 2023 Jul 12;13:11268. doi: 10.1038/s41598-023-38212-8 (PMC10338466; doi:10.1038/s41598-023-38212-8)
Supplement: Supplementary file 1 — Supplementary Information 1. [file 41598_2023_38212_MOESM1_ESM.pdf]

Supplementary Information

OrganoidChip facilitates hydrogel-free immobilization for fast and blur-free imaging of organoids

Khashayar Moshksayan [1,†], Anirudha Harihara [1,†], Sudip Mondal [1], Evan Hegarty [1], Todd Atherly [3], Dipak K. Sahoo [3], Albert E. Jergens [3], Jonathan P. Mochel [4], Karin Allenspach [3], Janet Zoldan [2] and Adela Ben-Yakar [1,2,*]

[1]Department of Mechanical Engineering, University of Texas at Austin, Austin, TX

[2]Department of Biomedical Engineering, University of Texas at Austin, Austin, TX

[3]Department of Veterinary Clinical Sciences, Iowa State University, Ames, IA

[4]Department of Biomedical Sciences, Iowa State University, Ames, IA

[†]These authors contributed equally to this work.

* Corresponding author, ben-yakar@mail.utexas.edu.

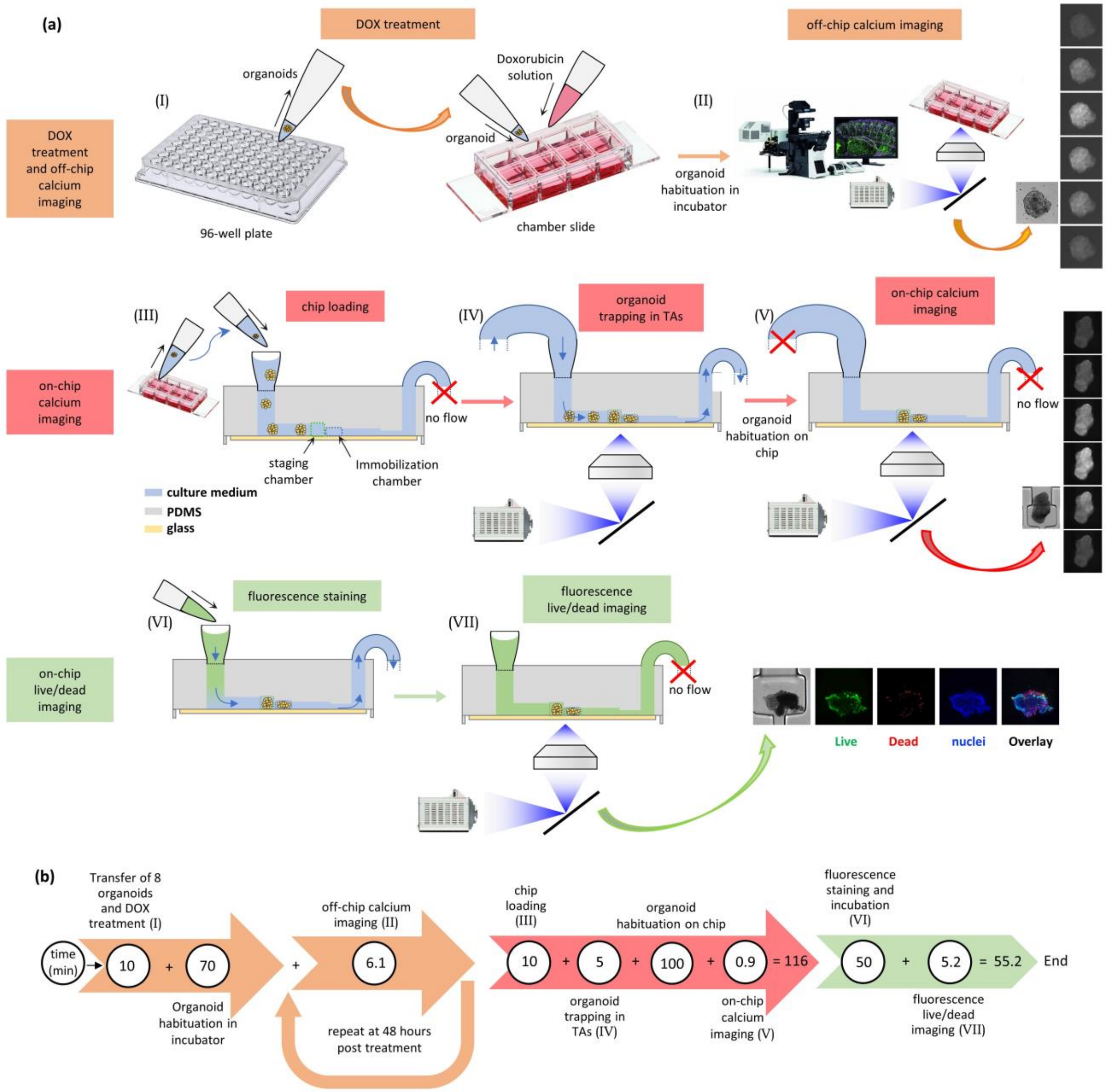

Figure S1: Flow chart for DOX treatment and imaging of cardiac organoids and the time dedicated to each step. (a) Various steps (I to VII) are required for imaging eight cardiac organoids on a chamber slide and on 6 TAs of the OrganoidChip. This figure consists of three parts, i.e., off-chip calcium imaging (1st row), on-chip calcium imaging (2nd row), and on-chip live/dead imaging (3rd row), each color-coded with orange, red, and green, respectively. The steps in each row are followed by the next row from left to right, up to the fluorescence live/dead imaging. (I) pipetting the cardiac organoids from the 96-well plate into the 8-well chamber slide and DOX treatment in a biosafety cabinet. (II) After incubating the organoids at 37 °C and 5% CO_2 overnight, calcium imaging was performed on an inverted microscope. The imaging was repeated at 48 hours after the drug treatment. (III) After 48 hours of DOX treatment, the organoids were transferred from the chamber slide onto the chip by pipetting them to the glass barrel mounted on the chip inlet. (IV) the organoids were distributed between the TAs with or without the assistive procedures. (V) Time-lapse calcium transients imaging was performed on organoids after 100 minutes of habituation on the chip. (VI) After calcium imaging, the tubing connected to the glass barrel was removed and the media inside the barrel was replaced with media containing Calcein AM, EthD-1, and Hoechst, and was incubated for 50 minutes. The flow inside the chip was established hydrostatically for 60 seconds, at 10-minute intervals, at 16 µl/min to expose the organoids to the fresh dye from the reservoir. (VII) The organoids were imaged with widefield fluorescence and confocal microscopes. (b) The time each

step in (a) requires in minutes. The times were measured during the experiment based on using eight cardiac organoids for the off- and on-chip imaging.

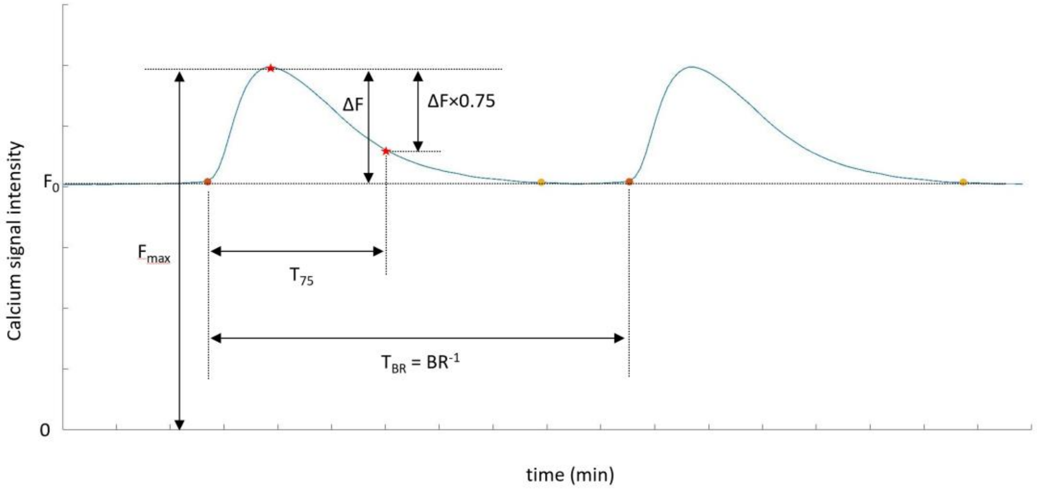

Figure S2: The schematic definition of the two parameters, T_{75} and $\Delta F/F_0$. T_{75} is the time duration from the calcium signal initiation (red dot) to the time when there is a 75% decay from the maximum calcium intensity signal. F_0 is the calcium signal intensity of the signal initiation time-point (red dot). BR is the beating rate. T_{BR} is the beating period.

Fast stage movements induce blur in the off-chip images

To further show the advantage of the OrganoidChip for blur-free imaging, we performed fast imaging of intestinal organoids in a 24-well plate after digesting the Matrigel to ensure they are no longer anchored. The organoids were subjected to significant agitation caused by the automated fast stage movements. We tried exposure times of 10, 40, 70 and 100 ms using widefield brightfield modality. The results showed that larger exposure times cause blur in the brightfield images of larger organoids if we do not wait for them to settle down (Fig. S3). Exposure times higher than 50 ms are common for imaging samples with low fluorescence intensity, therefore, fast imaging without proper immobilization can be very challenging especially for high-throughput applications.

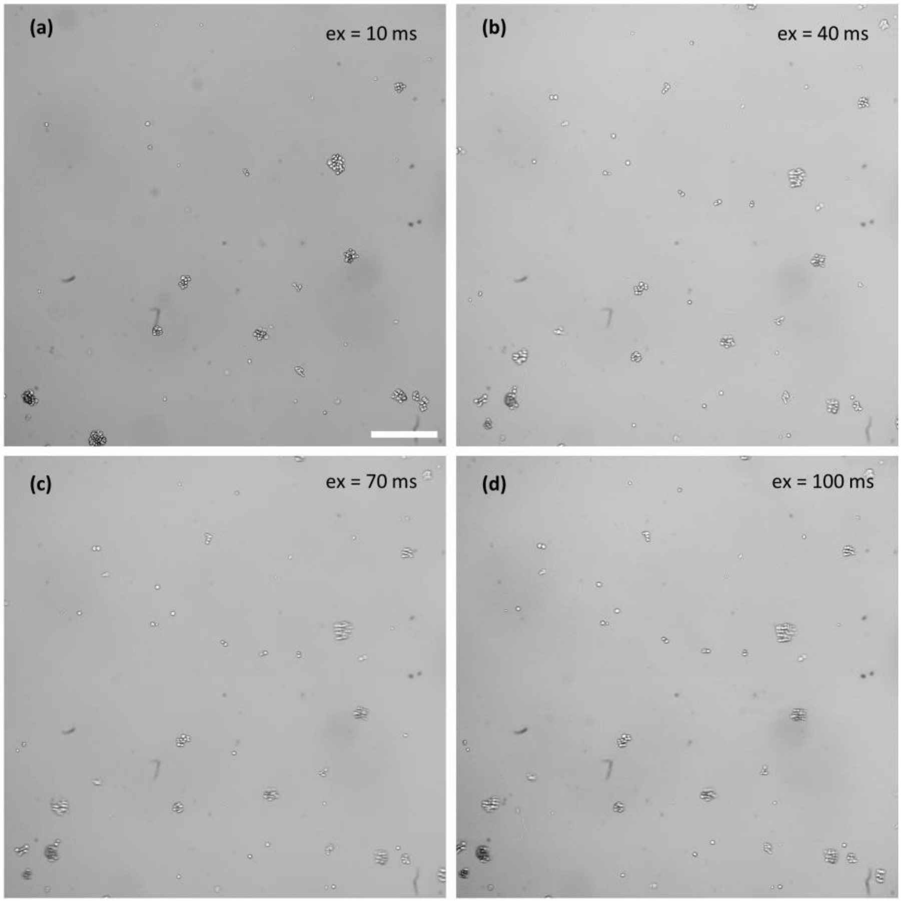

Figure S3: shows image blur due to the stage-induced organoids movements. Fast imaging necessitates fast stage movements which cannot be facilitated properly without immobilization. Figures a, b, c, and d correspond to exposure times of 10, 40, 70, and 100 ms, respectively. Scale bar represents 200 µm.

Connecting narrow channel design

We designed and tested two different versions of the OrganoidChip with similar geometries but with different lengths of the narrow channels (370 and 100 µm) that connect the ICs with the exit chamber. Although the lengths of the narrow channels were different, the difference in the induced hydraulic resistances caused by the them was negligible compared to the total hydraulic resistance of the entire chip as the majority of the resistance comes from the serpentine exit channel with a 100 µm height and a 71 mm length. The main difference between the two designs was that the chip with longer narrow channels (Fig. S4a) was able to keep organoids as small as 300 µm diameter in the ICs. However, the chip with shorter narrow channels (Fig. S4b) was not even capable of preventing the escape of large organoids of ~ 500 µm in diameter (Fig. S4b). The large organoids were able to escape the short narrow channels likely because of the lack of friction on them. Once a portion of an organoid exits the narrow channels, shown in Fig. S4b, the acting friction in the narrow channel is not sufficient to provide resistance against further movement of the organoid compared to the design in Fig. S4a. Therefore, for most of our experiments, we used chips with the longer narrow channels.

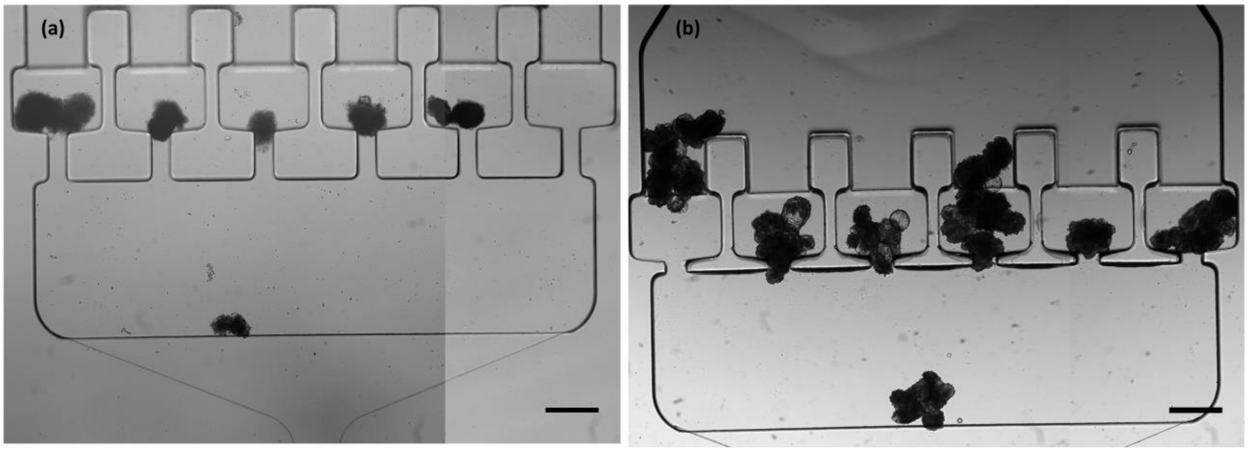

Figure S4: OrganoidChip designs with narrow channels of two different lengths, depicting organoids escaping the ICs of the chip designs. (a) Chip with long length of the narrow channels shows only a small size organoid escaping the IC. (b) Chip with short length of the narrow channels shows even a large size organoid escaping the IC. Both images were made from two images stitched together. The scale bars represent 400 µm.

Compression does not affect cardiac organoids' beating rate

A 4% agar solution was prepared by dissolving 4 g of Agar powder (Sigma-Aldrich) in 100 ml of deionized water, followed by autoclaving the mixture. The solution was aliquoted and stored for use. The agar pad was fabricated first by creating the desired height on a glass slide by stacking pieces of surgical tape on the two sides of the slide. Then, 1 ml of the agar solution was melted and added to the space between the tapes and was covered with a cover glass and was allowed to solidify. The cover glass was removed, and the agar pad was cut according to Fig. S5a to keep the connection and nutrient transport between the culture medium within the petri dish and the organoids upon sandwiching them with the cover glass. Then, the agar pad and the organoids were transferred to the petri dish and habituated for 70 minutes in the mini-incubator. The organoids' beating rate were measured at various time-points before and after placing the cover glass, 47 minutes after the habituation was finished (Fig. S5).

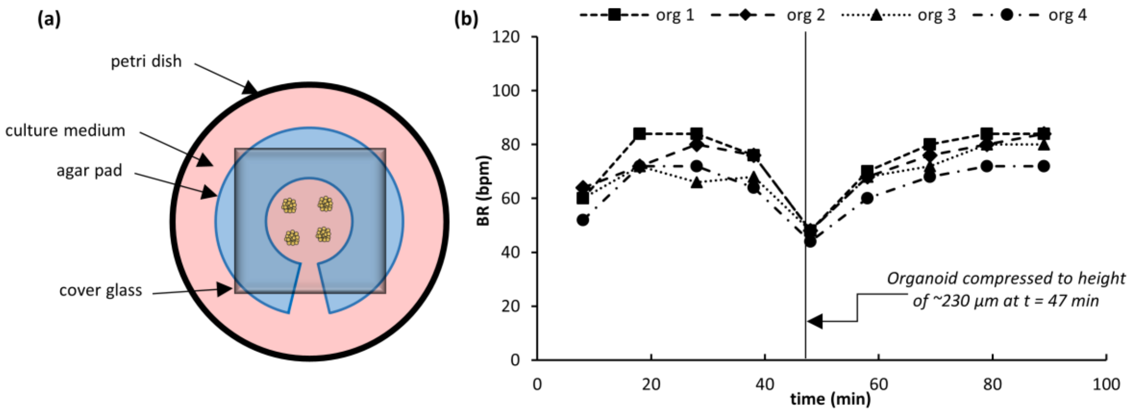

Figure S5: off-chip compression of cardiac organoids. (a) schematic shows the experimental setup consisting of a petri dish, an agar pad with a height of 230 µm, and a cover glass that covers the top of the agar pad, bringing the height of organoids to 230 µm. (b) beating rate monitoring of four organoids before and after the compression. The time begins after the habituation period.

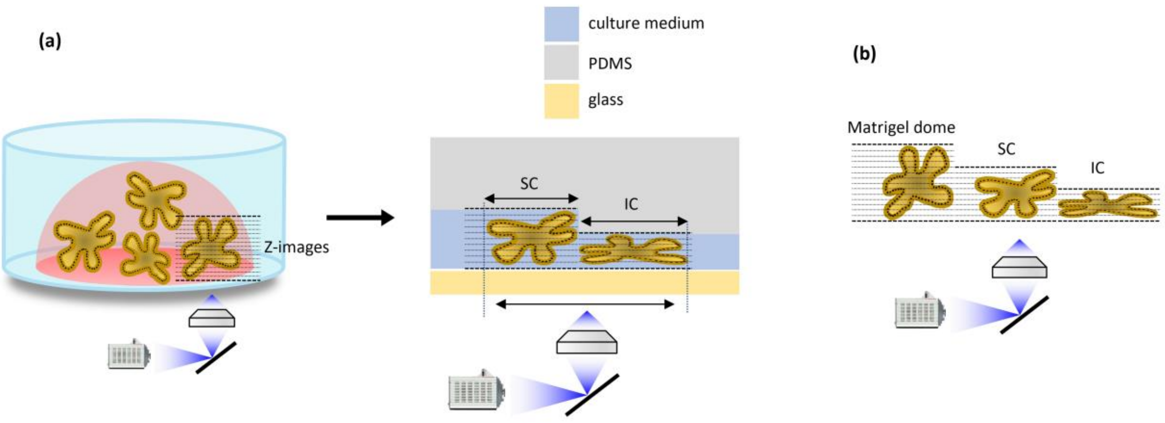

Figure S6: The number of z-images required to image the entire organoid is reduced in the OrganoidChip. (a) the schematic illustrates the difference in the shape of an intestinal organoid between when it is within the Matrigel dome and in the TAs of the chip. The z-images are shown with horizontal dashed lines. (b) while the distance between the z-images is the same, the number of z-images required for the Matrigel dome is significantly higher than when the organoid resides in the TAs. This will reduce the number of images, the storage space for them and the total imaging time.

Equation S1: The sigmoidal Imax model used for fitting the dose-response curves in the OriginPro software. The parameters for each curve are displayed in Table S2.

$$y = A_1 + \frac{A_2 - A_1}{1 + 10^{(log x_0 - x)*p}} \qquad (eq.S1)$$

Table S1: Tabulates the quantities of time, speed of spin coating, UV energy and temperature used for the photolithography of the mold.

	SPIN COATING TIME AND SPEED	SOFT BAKE 1 AND 2, TIME AND TEMPERATURE	EXPOSURE TIME AND ENERGY	POST EXPOSURE BAKE 1 AND 2, TIME AND TEMPERATURE	DEVELOPING TIME
**LAYER 1: SU8-2050**	60 s, 1700 rpm	5 min, 65 °C then 15.5 min, 95 °C	19.2 s, 230 mw/cm^3	1- 4 min, 65 °C<br>2- 9 min, 95 °C	9 min
**LAYER 2: SU8-2100**	7s, 500 rpm then 33 s, 1175 rpm	7 min, 65 °C then 60 min, 95 °C	29.5 s, 370 mw/cm^3	1- 5 min, 65 °C<br>2- 20 min, 95 °C	NA
**LAYER 3: SU8-2100**	7s, 500 rpm then 33 s, 1850 rpm	7 min, 65 °C then 52 min, 95 °C	29.7 s, 370 mw/cm^3	1- 5 min, 65 °C<br>2- 20 min, 95 °C	30 to 45 min

Table S2: The regression parameters used for the sigmoidal Imax model presented in equation S1.

	Constants			
**Dose-response parameter**	A1	A2	LOGx_0 (center)	p (Hill slope)
Cardiac viability – off-chip	0.049	1.085	0.656	-1.345
Cardiac viability – on-chip	0.029	1.096	0.663	-1.976
Intestinal viability – off-chip	0.088	224.660	-2.051	-1.180
Intestinal viability – on-chip	0.019	334.571	-1.550	-1.634
BR – off-chip	-0.935	0.172	0.598	-2.600
BR – on-chip	-0.992	-0.132	0.543	-2.134
$\Delta F/F_0$ – off-chip	-0.986	-0.074	0.653	-3.285
$\Delta F/F_0$ – on-chip	-0.998	-0.148	0.719	-3.641
FBT_{75}– off-chip	-0.989	-0.115	0.673	-3.716
FBT_{75}– on-chip	-0.994	-0.234	0.657	-2.298
BT_{75}– off-chip	-0.958	0.039	0.753	-2.755
BT_{75}– on-chip	-1.012	0.741	0.141	-0.752

Table S3: The IC50 for each fractional beating parameter and viability of cardiac organoids is shown along with the standard error (SE).

Parameter	$IC_{50,off\text{-}chip}$ ± SE	$IC_{50,on\text{-}chip}$ ± SE
Δ(BR)	0.60 ± 0.00	0.54 ± 0.10
$\Delta(\Delta F/F_0)$	0.65 ± 0.39	0.72 ± 0.54
$\Delta(BT_{75})$	0.75 ± 0.25	0.71 ± 0.20
$\Delta(FBT_{75})$	0.67± 0.53	0.66± 0.53
Viability	0.75 ± 0.30	0.66 ± 0.05

Table S4: The F-tests performed to compare the fitted dose-response curves of the off- vs. on-chip conditions. They all show no statistically significant difference.

Organoid type	Parameter	Statistically Different?	P-value
Cardiac	BR	No	0.14
	BT_{75}	No	0.57
	$\Delta F/F_0$	No	1.00
	FBT_{75}	No	0.95
	Viability	No	0.92
Intestinal	Viability	No	0.92

Table S5: The P-values corresponding to the F-tests performed to compare the fitted dose-response curves of various beating kinetics parameters and viability against each other. The curves of each condition, i.e., the off- (a) and on-chip (b), were compared separately. They all show no statistically significant difference.

(a)

Off-chip	BR	$\Delta F/F_0$	T75xBR	FBT75	viability
BR	-	0.81	0.91	0.79	0.44
$\Delta F/F_0$	-	-	0.90	1.00	0.89
T75xBR	-	-	-	0.89	0.66
FBT75	-	-	-	-	0.89

(b)

On-chip	BR	$\Delta F/F_0$	T75xBR	FBT75	viability
BR	-	0.87	0.61	1.00	0.09
$\Delta F/F_0$	-	-	0.77	0.95	0.79
T75xBR	-	-	-	0.77	0.67
FBT75	-	-	-	-	0.46

OrganoidChip reduces the required time for a high-throughput imaging session

We measured the amount of time that is required for each step of the calcium and live and dead imaging of a single cardiac organoid on a chamber slide and the OrganoidChip using an inverted microscope and a 10× objective. Using the 10× objective, two TAs each containing at least one organoid can be imaged simultaneously. Then, the times required for a chamber slide and an OrganoidChip experiment, each having six organoids, were calculated (Table S6). Moreover, we anticipate that according to the last column of table S6, the calcium imaging and the live and dead imaging time can be reduced by an order of magnitude when imaging 96×6 organoids on a high-throughput 96-well OrganoidChip compared to the conventional flat-bottom 96-well plates. *

Table S6: Time required for each step performed on a chamber slide and on the chip for 6 organoids. The last column presents the time that would take when imaging 96×6 organoids on a 96-well OrganoidChip compared to on chamber slides. Numbers marked by * indicate that the time associated with that step was considered only once, and not 96 times, for calculating the t_{total} in the last column.

		finding the organoids (s)	changing to high-NA objective (s)	finding ROIs and focal planes (s)	introducing and removing the dyes (s)	setting camera parameters (s)	bright-field imaging (s)	fluorescence imaging (s)	total time for imaging 6 organoids (s)	t_{total} (hour)
calcium imaging	$t_{chamber\ slide}$	90	30	30	-	35*	30	60	275	7.2
	t_{chip}	0	0	0	-	10*	15	30	55	1.2
live and dead imaging	$t_{chamber\ slide}$	90	30	120	360	150*	14	29	792	18.0
	t_{chip}	0	0	20*	120*	125*	8	16	290	0.8
